# Stepped-wedge randomized controlled trial of laparoscopic ventral mesh rectopexy in adults with chronic constipation

**DOI:** 10.1007/s10151-022-02633-w

**Published:** 2022-05-19

**Authors:** U. Grossi, J. Lacy-Colson, S. R. Brown, S. Cross, S. Eldridge, M. Jordan, J. Mason, C. Norton, S. M. Scott, N. Stevens, S. Taheri, C. H. Knowles

**Affiliations:** 1 Centre for Neuroscience, Surgery and Trauma, Blizard Institute, Barts and the London School of Medicine and Dentistry, Queen Mary University of London, London, UK; 2 Department of Surgery, Oncology and Gastroenterology, DISCOG, University of Padua, Padua, Italy; 3 Royal Shrewsbury Hospital, Shrewsbury and Telford Hospital NHS Trust, Shrewsbury, UK; 4 Sheffield Teaching Hospitals NHS Trust, Sheffield, UK; 5 School of Health and Related Research, University of Sheffield, Sheffield, UK; 6 Pragmatic Clinical Trials Unit, Institute of Population Health Sciences, Barts and the London School of Medicine and Dentistry, Queen Mary University of London, London, UK; 7 Warwick Clinical Trials Unit, Warwick Medical School, University of Warwick, Coventry, UK; 8 Faculty of Nursing, Midwifery and Palliative Care, King’s College London, London, UK

**Keywords:** Constipation, Stepped wedge, Randomized controlled trial, Rectopexy, Rectal prolapse

## Abstract

**Background:**

The effectiveness of laparoscopic ventral mesh rectopexy (LVMR) in patients with defecatory disorders secondary to internal rectal prolapse is poorly evidenced. A UK-based multicenter randomized controlled trial was designed to determine the clinical efficacy of LVMR compared to controls at medium-term follow-up.

**Methods:**

The randomized controlled trial was conducted from March 1, 2015 TO January 31, 2019. A stepped-wedge RCT design permitted observer-masked data comparisons between patients awaiting LVMR (controls) with those who had undergone surgery. Adult participants with radiologically confirmed IRP refractory to conservative treatment were randomized to three arms with different delays before surgery. Efficacy outcome data were collected at equally stepped time points (12, 24, 36, 48, 60, and 72 weeks). Clinical efficacy of LVMR compared to controls was defined as ≥ 1.0-point reduction in Patient Assessment of Constipation-Quality of Life and/or Symptoms (PAC-QOL and/or PAC-SYM) scores at 24 weeks. Secondary outcome measures included 14-day diary data, the Generalized Anxiety Disorder scale (GAD-7), the Patient Health Questionnaire-9 (PHQ-9), St Marks incontinence score, the Pelvic Organ Prolapse/Urinary Incontinence Sexual Questionnaire (PISQ-12), the chronic constipation Behavioral Response to Illness Questionnaire (CC-BRQ), and the Brief Illness Perception Questionnaire (BIPQ).

**Results:**

Of a calculated sample size of 114, only 28 patients (100% female) were randomized from 6 institutions (due mainly to national pause on mesh-related surgery). Nine were assigned to the T0 arm, 10 to T12, and 9 to T24. There were no substantial differences in baseline characteristics between the three arms. Compared to baseline, significant reduction (improvement) in PAC-QOL and PAC-SYM scores were observed at 24 weeks post-surgery (– 1.09 [95% CI – 1.76, – 0.41], *p* = 0.0019, and – 0.92 [– 1.52, – 0.32], *p* = 0.0029, respectively) in the 19 patients available for analysis (9 were excluded for dropout [*n* = 2] or missing primary outcome [*n* = 7]). There was a clinically significant long-term reduction in PAC-QOL scores (− 1.38 [− 2.94, 0.19], *p* = 0.0840 at 72 weeks). Statistically significant improvements in PAC-SYM scores persisted to 72 weeks (− 1.51 [− 2.87, − 0.16], *p* = 0.0289). Compared to baseline, no differences were found in secondary outcomes, except for significant improvements at 24 and 48 weeks on CC-BRQ avoidance behavior (− 14.3 [95% CI − 23.3, − 5.4], and − 0.92 [− 1.52, − 0.32], respectively), CC-BRQ safety behavior (− 13.7 [95% CI − 20.5, − 7.0], and − 13.0 [− 19.8, − 6.1], respectively), and BIPQ negative perceptions (− 16.3 [95% CI − 23.5, − 9.0], and − 10.5 [− 17.9, − 3.2], respectively).

**Conclusions:**

With the caveat of under-powering due to poor recruitment, the study presents the first randomized trial evidence of short-term benefit of LVMR for internal rectal prolapse.

**Trial registration:**

ISRCTN Registry (ISRCTN11747152).

**Supplementary Information:**

The online version contains supplementary material available at 10.1007/s10151-022-02633-w.

## Introduction

Dynamic structural abnormalities of the anorectum and pelvic floor can cause symptoms of obstructed defecation and fecal incontinence [1], and are found in an important subgroup of patients with chronic constipation [2, 3]. The most common abnormalities (either singly or together) are rectocele and intussusception [3, 4]. While parameters for diagnosis and intervention vary [1, 5, 6], structurally significant rectoceles and high-grade intussusception (i.e. those descending to the level of anal canal or beyond) [1] may benefit from surgical repair in well-selected patients. Procedures broadly aim to reinforce the rectovaginal septum (mainly focused on rectocele) [7–9], excise part of the rectal wall (most commonly using stapling devices) [10–12], or suspend the rectum (mainly forms of rectopexy) [13, 14]. The varying popularity of numerous procedures to address these problems reflects the fact that no single approach has achieved obvious clinical primacy and also that there is no high-quality evidence base for decision-making [15].

Laparoscopic ventral mesh rectopexy (LVMR) was first described for external rectal prolapse (ERP) in 1992 [16], and has progressed into international practice as a relatively safe, minimally invasive approach to internal rectal prolapse (IRP) with or without rectocele [17–20]. While some large patient series provide general support for LVMR in populations of patients with a mix of symptomatic presentations (obstructed defecation or incontinence) due to ERP or IRP [17–19, 21, 22], the utility of LVMR for patients with obstructed defecation and IRP is not well-supported by published evidence. Indeed, a previous UK National Institute for Health Research-funded systematic review included only 18 studies with a total of 1238 patients [23]. Of these, the vast majority of included studies provided only level IV (Oxford) evidence. Furthermore, outcomes have generally been based on poorly validated measures, e.g. patient global rating scales and the obstructed defecation syndrome (ODS) score [24–26], which were originally developed to evaluate the effect of surgery [27, 28]. There is concern that objectively determined long-term outcomes of LVMR using validated measures will not match those from enthusiasm-driven case series (as has been the case for numerous other surgical procedures for chronic constipation) [29], and this question has become more important with the international scrutiny of mesh-related complications in general. [30].

Therefore, a UK-based multicenter randomized controlled trial (RCT) was designed to determine the clinical efficacy of LVMR compared to controls at short-term follow-up (24 weeks). Secondary objectives were to determine: (1) the clinical effectiveness of LVMR in the medium-term (to 48 weeks to a maximum of 72 weeks), and (2) preoperative determinants of outcome. A detailed description of the study protocol was published elsewhere. [31].

## Materials and methods

### Patients

This UK multi-institutional RCT was conducted from March 1, 2015, to January 31, 2019, as part of a UK National Institute of Heath Research-funded programme grant (PGfAR: RP-PG-0612-20001) aimed at developing the evidence base for the management of chronic constipation in adults, which is currently lacking [32].

A stepped-wedge randomized trial design permitted observer-masked data comparisons between patients awaiting intervention with those who had undergone surgery. Contrary to most stepped-wedge trials individual patients were randomized rather than utilizing cluster sampling. This is, in effect, a modification of a standard parallel-group, waiting-list control design, but with several advantages. First, a stepped-wedge design is more efficient and thus improves recruitment feasibility (the bane of nearly all surgical trials). Simulation demonstrated that a parallel-group design required a much larger sample size than that proposed for the current study at the same power. Second, the trial design meant that there was only a one-in-three chance (rather than one-in-two chance for a parallel group) of waiting 6 months for surgery, which was more acceptable to patients. The study received national ethical approval (15/LO/0609) and all patients provided their written informed consent. The study was registered with the ISRCTN Registry (ISRCTN11747152 [https://doi.org/10.1186/ISRCTN11747152]).

Eligibility criteria were: (1) age 18–70 years; (2) self-report of problematic constipation; (3) symptom onset > 6 months prior to recruitment; (4) symptoms meeting the American Gastroenterological Association definition of constipation [33]; (5) refractory constipation after a minimum basic standard (lifestyle and dietary measures and ≥ 2 laxatives or prokinetics) tried with no resolution of symptoms and no time requirement; (6) ability to understand written and spoken English (due to questionnaire validity); (7) ability and willingness to give informed consent; (8) failure of non-surgical interventions (minimum of nurse-led behavioral therapy) [34]; (9) IRP as determined by clinical examination and defecography, using the following criteria: (a) recto-anal or recto-rectal intussusception ± other dynamic pelvic floor abnormalities (e.g. rectocele, enterocele, excessive perineal descent); (b) deemed to be obstructing and/or associated with protracted or incomplete contrast evacuation by expert review. [35].

Exclusion criteria were: (1) significant organic colonic disease (red flag symptoms, e.g. rectal bleeding not previously investigated), inflammatory bowel disease, megacolon or megarectum (if diagnosed beforehand), severe diverticulosis/stricture/birth defects deemed to contribute to symptoms; (2) major colorectal excisional surgery; (3) current overt pelvic organ (bladder, uterus, and/or external rectal) prolapse or disease requiring obvious surgical intervention other than LVMR; (4) previous rectopexy; (5) sacral nerve stimulator in situ; (6) rectal impaction (as defined by digital and abdominal examination); (7) significant neurological disease (e.g. Parkinson’s disease, spinal injury, multiple sclerosis, diabetic neuropathy); (8) significant connective tissue disease (e.g. scleroderma, systemic sclerosis, systemic lupus erythematosus [not hypermobility alone]); (9) significant medical comorbidities and activity of daily living impairment (Barthel index ≤ 11); (10) major active psychiatric diagnosis (e.g. schizophrenia, major depressive illness and mania); (11) chronic regular opioid use (at least once daily) deemed to be the cause of constipation based on temporal association of symptoms with onset of therapy; (12) pregnancy or intention to become pregnant during study period; (13) known severe intra-abdominal adhesions.

Final review by pelvic floor multidisciplinary decision team (as per National Health Service [NHS] England recommendation) [36] to confirm appropriateness for surgery was performed for all patients.

### Randomization and masking

**Fig. 1:**
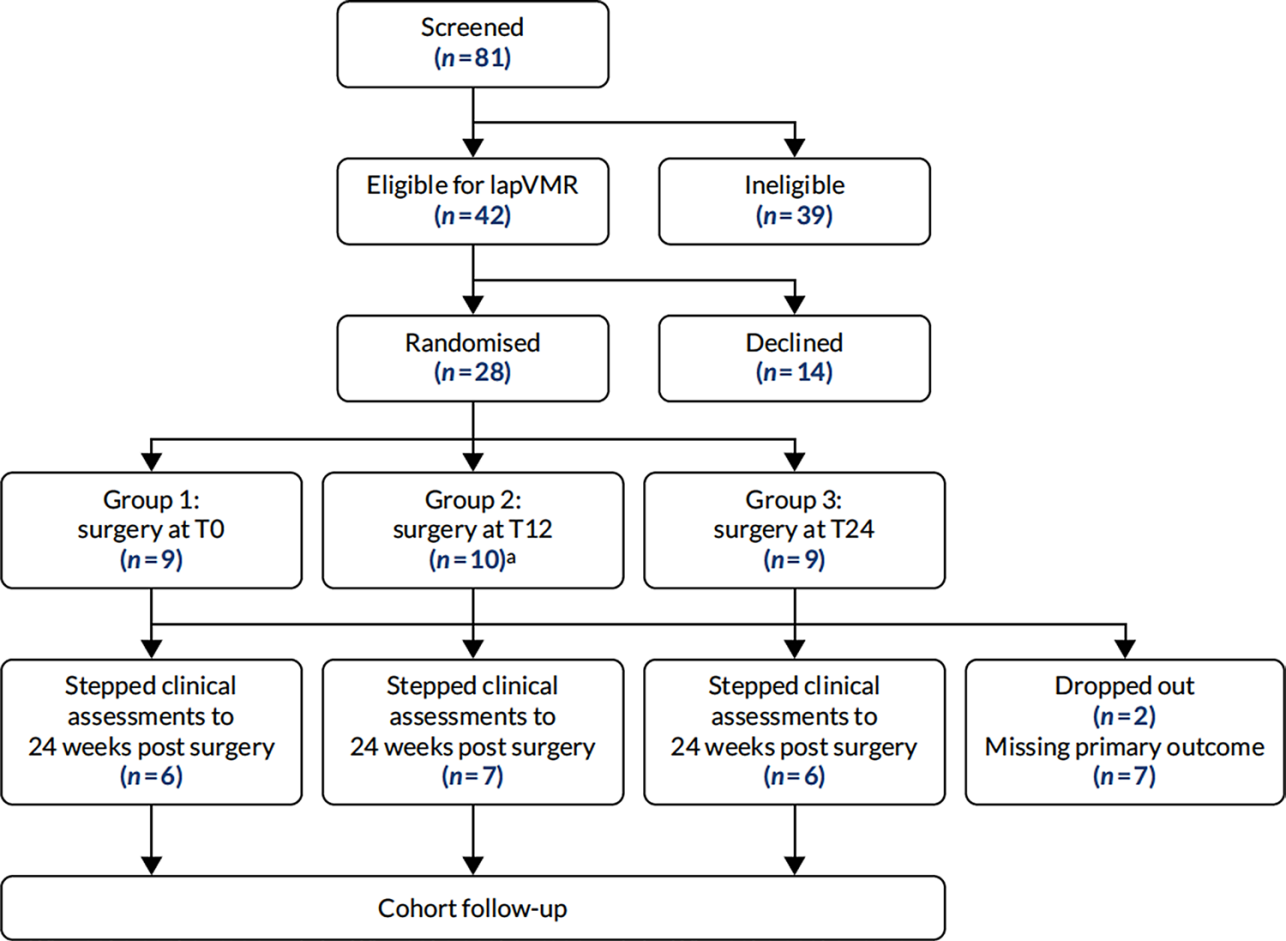
The CapaCiTY trial 3 Consolidated Standards of Reporting Trials (CONSORT) flow diagram. One patient did not undergo surgery; this patient continued to participate and was included in analysis on intention-to-treat principles

Participants were randomized to three arms with different delays before surgery (Fig. 1). In group I, LVMR was performed at T0; in group II, at 12 weeks (T12); in group III, at 24 weeks (T24). In all arms, there was a period of 4 weeks post-eligibility screening to arrange the logistics of surgery and ensure that patients had returned to their normal life routine after various assessments. Randomization was stratified by center. The Pragmatic Clinical Trials Unit (PCTU) at Queen Mary University of London developed a validated online randomization system, which was accessed by suitably trained and delegated researchers at recruiting sites and followed the PCTU-approved standard operating procedure for the study.

Patients and clinicians were necessarily aware of allocation to different waiting times. However, to minimize bias, a blinded researcher collected outcome data. For quantitative analysis, an analysis plan was developed and signed off by investigators and statisticians who were blind to allocation status and index intervention.

### Intervention

LVMR was performed according to a standard technique [31, 37, 38], starting with a peritoneal incision at the level of the sacral promontory and extending caudally (avoiding the hypogastric nerves along the side of the mesorectum) to the deepest part of the pouch of Douglas, and continued down the rectovaginal septum to the pelvic floor. The mesh was sutured to the ventral aspect of the distal rectum and further fixed to the lateral seromuscular borders of the rectum proximal and distal to the incised pouch of Douglas ± pelvic floor. If deemed necessary, the posterior vaginal fornix was elevated and sutured to the anterior aspect of the mesh to allow closure of the rectovaginal septum and correction of a mid-compartment prolapse (if present). The type of mesh inserted was left to surgeon’s choice (not being dependent on any specific clinical grounds). All participating surgeons had performed a minimum of 50 LVMR previously.

Surgery was performed as a day case or short stay procedure [39]. Postoperative management followed routine clinical care. Laxative use was standardized to a weaning course of macrogol transdermal delivery system (TDS) immediately postoperatively for 1 day, then reduced according to ease of bowel movements.

### Surgical quality assessment

Adherence to the agreed procedural technique for the first included patient from each center was independently remotely assessed by a delegated surgical team provided by the Pelvic Floor Section of the Association of Coloproctology of Great Britain and Ireland. Monitoring and quality control were conducted remotely via video submission and assessed against the standardized LVMR protocol and defined assessment criteria [31, 37, 38]. Monitoring took the form of planned, random and triggered sessions (Supplementary Table 1).

### Outcomes

The primary clinical outcome was Patient Assessment of Constipation Quality of Life (PAC-QOL) score [40]. This widely used, psychometrically robust measure of overall treatment response with concurrent validity to patient global ratings of success has been used by previous behavioral therapies and surgical trials, including LVMR [41], in chronic constipation [42]. For a chronic condition such as chronic constipation, a difference of 1.0 point in the primary outcome (score range = 1–4, with higher scores meaning higher negative effects on quality of life) was considered clinically important and also the minimum required to justify the cost and invasive nature of LVMR, or of a more complex and expensive treatment [43].

Secondary outcomes measures included 14-day diary data prior to each assessment (to record bowel frequency, whether each evacuation was ‘spontaneous’ [no use of laxatives] and/or ‘complete’, concurrent medication, health contacts, time away from normal activities including work, since the patient’s last visit), Generalized Anxiety Disorder scale (GAD-7) [44], Patient Health Questionnaire-9 (PHQ-9) [45], St Marks incontinence score [46], Pelvic Organ Prolapse/Urinary Incontinence Sexual Questionnaire (PISQ-12) [47], avoidant and ‘all or nothing’ behavior subscales of the chronic constipation Behavioral Response to Illness Questionnaire (CC-BRQ) [48], the Brief Illness Perception Questionnaire (BIPQ) [49], the EuroQol Visual Analogue Scale (EQ-VAS), the EuroQol Health Outcome Measure (EQ-5D-5L) [50], and the global patient satisfaction/improvement score on a five-point Likert scale. LVMR has a number of specific complications in addition to the general risks of surgery. These were recorded for outcome reporting. The study (not being of a medicinal product) did not record unrelated adverse events.

Participant, surgeon and research staff experience was investigated through individual digitally recorded telephone or in-clinic interviews up to 1 year after surgery with a purposively selected sample to represent a range of demographics. Separate consent was taken for interviews. Data were analyzed using a pragmatic thematic and qualitative analysis.

### Follow-up

The study duration allowed for follow-up to a maximum of 96 weeks (i.e. 24 months) with data collection at 0, 12, 24, 36, 48 weeks post run in (stepped wedge) and thence at 12-week intervals within the cohort assessments at 60, 72, 84 and 96 weeks post run in. Thereafter, participants left the study and returned to ‘routine clinical care’ as determined within their local National Health Service institution (or were recruited to subsequent trials).

### Statistical analysis

The sample size was calculated using the primary clinical outcome [40] by simulation using the ‘simsam’ package in Stata^®^ V.14.2 (Stata Corporation, College Station, Texas, USA). Using a stepped wedge design, we hypothesized that PAC-QOL score at any time point during follow-up will be approximately 1.0 point lower (better) than preoperative participants. We assumed that PAC-QOL score followed a normal distribution over all time points with a standard deviation (SD) of 1.5 points and with a correlation between repeated assessments equal to 0.5 points. Simulation showed that detection of a 1-point difference in PAC-QOL score at 6 months with 95% power (purposely chosen to reflect the magnitude and risk of intervention) at the 5% significance level required 34 participants in each of the three arms. Allowing for a 10% loss to follow-up, a sample size of 38 was needed per arm, for a total sample size of 114 patients across the 3 arms. Should the correlation between repeated assessments be < 0.5 points, a sample size of 114 will still provide at least 90% power for the study. This was calculated using the same simulation procedure with correlations of 0.3 and 0.1 points.

The primary outcome was analyzed as a continuous variables on intent-to-treat basis at 24 weeks post-surgery. PAC-QOL scores at the time-points T0, T12, T24, T36, T48, T60, and T72 weeks post run in period in the three arms were analyzed using a mixed linear regression model, adjusting for a random effect of participant and a fixed effect of time since randomization, to estimate mean differences between PAC-QOL score before and after LVMR. To model the effects of surgery, dummy variables were used to indicate if participants had already received treatment before each follow-up time. The Kenward–Roger correction was employed to account for inflated type I error rates due to the small sample size. The contrast of primary interest was between the score at 24 weeks after surgery and the score at baseline. Some outcomes were scores calculated by summing the responses to all the questions in a questionnaire. If fewer than half of the questions were unanswered the missing responses were imputed with the mean of the available cases. All outcomes were analyzed under a ‘missing at random’ assumption (i.e. assuming that ‘missingness’ depended only on outcomes that had been observed). Patient Assessment of Constipation‐Symptoms (PAC-SYM) scores [51] were analyzed by the same approach as above. Binary outcomes were summarized at 24 and 48 weeks post-surgery with number and percent indicating problems and an odds ratio comparing 24 and 48 week outcomes to baseline. Data were analyzed using Stata^®^ V.14.2 (Stata Corporation, College Station, Texas, USA).

## Results

From March 1, 2016, to January 31, 2019, of 42 eligible patients, 28 (100% females) were randomized from 6 UK institutions (Fig. 1), representing a significant under-recruitment of the 114 patients required by sample size calculation. The main reason for poor recruitment was the evolution of the mesh controversy during the trial [30], which stopped many centers from delivering the procedure and many patients coming forward for surgery.

Of the 28 patients, 9 were assigned to the T0 arm, 10 to the T12, and 9 to the T24 arm (Fig. 1). The characteristics of the 28 patients are presented in Table 1. There were no substantial differences in baseline characteristics between trial arms. The outcome measures at baseline, collected before the 4-week run in period, are summarized in Supplementary Table 2. Biologic mesh was used in three patients (one patient per group).

**Table 1:** Demographic and clinical characteristics of randomized patients

	Group 1 (T0) (*n* = 9)	Group 2 (T12) (*n* = 10)	Group 3 (T24) (*n* = 9)
Age (years)^a^	59 (39–66)	56 (42–64)	55 (49–58)
Comorbidities (*n*, %)	7 (78)	9 (90)	6 (67)
Cardiovascular	4 (44)	3 (30)	1 (11)
Respiratory	0 (0)	2 (20)	1 (11)
Gastrointestinal	5 (56)	3 (30)	2 (22)
Metabolic	0 (0)	3 (30)	4 (44)
Hematological	2 (22)	1 (10)	0 (0)
Genito-urinary	2 (22)	0 (0)	2 (22)
Neurological	2 (22)	4 (40)	3 (33)
Psychiatric	2 (22)	5 (50)	4 (44)
Dermatological	1 (11)	3 (30)	1 (11)
Musculoskeletal	3 (33)	2 (20)	3 (33)
Previous surgery (*n*, %)	5 (56)	10 (100)	7 (78)
Abdominal	2 (33)	3 (30)	2 (25)
Gynecological	4 (67)	9 (90)	5 (63)
Proctological and perineal	0 (0)	4 (40)	1 (13)
Duration of constipation symptoms (months)^b^	68.7 (36.9)	63.3 (31.6)	76.6 (55.4)
Sexual history (*n*, %)			
Sexually active	5 (63)	6 (60)	3 (33)
Female of child bearing potential	4 (57)	4 (40)	4 (44)
Over 1 year post-menopausal	3 (43)	6 (60)	4 (44)
Surgically sterile	3 (38)	5 (50)	4 (44)
Previous deliveries (*n*, %)	9 (100)	10 (100)	9 (100)
Vaginal deliveries^b^	2.1 (1.1)	2.5 (1.1)	2.7 (1.0)
Caesareans^b^	1.0 (1.1)	0.1 (0.3)	0.4 (1.0)
Forceps/ventose^b^	0.2 (0.7)	0.5 (0.7)	0.3 (0.7)
Episiotomy^b^	1.1 (1.1)	0.2 (0.4)	1.0 (1.2)
Obstetric tear^b^	0.6 (0.7)	0.4 (0.5)	0.6 (0.5)
Fecal incontinence symptoms (*n*, %)	7 (78)	9 (90)	7 (78)
Fecal urgency	4 (50)	8 (80)	5 (71)
Urge fecal incontinence	5 (63)	6 (60)	3 (43)
Passive fecal incontinence	4 (50)	6 (60)	3 (43)
Post defecation leakage	5 (63)	4 (40)	4 (57)
Difficulty in wiping clean	6 (75)	5 (50)	4 (57)
Vaginal bulging (*n*, %)	6 (67)	5 (50)	7 (78)

^a^Values are median (IQR)

^b^Values are mean (SD)

### Safety analyses

There were no conversions to open surgery. Thirty adverse events were reported by 16 patients, of which 20 were considered to have possible causality related to surgery (Supplementary Table 3); however, none had any long-term sequelae. There were five serious adverse events of which four were deemed to be related to surgery. Three of these were for postoperative pain (expected), one was for pneumonia and none resulted in long-term patient harm (Clavien–Dindo I). No patients had mesh erosions.

### Clinical effectiveness

Two patients dropped out of the study before the primary end point and a further 7 failed to complete the primary outcome, which was, therefore, completed by only 19 patients. There was a substantive reduction in estimated PAC-QoL score at 24 weeks compared with the baseline of 1.09 points (95% CI – 1.76, – 0.41], *p* = 0.0019), exceeding that sought by design (1.0 points). A similar magnitude of change was observed for the modelled secondary outcome (i.e. PAC-SYM score, – 0.92 [95% CI – 1.52, – 0.32], *p* = 0.0029) (Table 2). Reductions in scores were sustained at later time points, accepting a strong chance of attrition bias.

**Table 2:** Total PAC-QoL and PAC-SYM scores at baseline and follow-up points post-surgery, with 95% CI and *p* value for change from baseline to each follow-up point

	*N*	Mean	Change from baseline^a^	95% CI	*P* value
PAC-QOL total scores					
Baseline	26	2.63	–	–	–
12 weeks	23	1.35	– 1.04	– 1.54, – 0.55	0.0001
24 weeks	19	1.26	– 1.09	– 1.76, – 0.41	0.0019
36 weeks	19	1.47	– 0.98	– 1.87, – 0.10	0.0296
48 weeks	17	1.43	– 1.07	– 2.16, 0.02	0.0552
60 weeks	9	1.22	– 1.26	– 2.56, 0.05	0.0587
72 weeks	5	1.11	– 1.38	– 2.94, 0.19	0.0840
PAC-SYM total scores					
Baseline	26	2.24	–	–	–
12 weeks	23	1.15	– 0.97	– 1.41, – 0.53	< 0.0001
24 weeks	18	1.19	– 0.92	– 1.52, – 0.32	0.0029
36 weeks	19	1.25	– 1.03	– 1.80, – 0.26	0.0094
48 weeks	17	1.36	– 0.97	– 1.92, – 0.02	0.0444
60 weeks	9	1.19	– 1.16	– 2.28, – 0.03	0.0448
72 weeks	5	0.82	– 1.51	– 2.87, – 0.16	0.0289

*PAC-QOL* Patient Assessment of Constipation Quality of Life, *PAC-SYM* Patient Assessment of Constipation Symptoms

^a^Estimated changes (points) are adjusted for time

Secondary outcomes are shown in Table 3, with positive directional effects for nearly all outcomes, with some quite substantial improvements in measures, including > 25% scalar improvements in psychological measures (PHQ-9 score, GAD-7 score, St Mark’s Incontinence Score and EQ-VAS score). Global patient satisfaction was 2.7 points at 24 weeks (i.e. closest to ‘very satisfied’), although this dropped to 2.2 points (i.e. closest to ‘moderately satisfied’) at 48 weeks. This result was mirrored in the global patient improvement score (EQ-VAS score 0–100 points between ‘no effect’ and ‘complete cure’), which was 72.2 points at 24 weeks and 56.5 points at 48 weeks.

**Table 3:** Continuous secondary outcomes, with unadjusted estimate of difference in mean scores at 24 and 48 weeks post-surgery compared to baseline

	Time	*N*	Mean (SD)	Median (IQR)	Change from baseline (95% CI)
PAC-QOL score, dissatisfaction	Baseline	26	3.1 (0.6)	3.1 (2.8, 3.6)	Reference
	24 weeks	19	1.8 (1.0)	1.8 (1.0, 2.4)	– 1.3 (– 1.8, – 0.8)
	48 weeks	17	2.1 (1.0)	2.0 (1.4, 2.8)	– 1.0 (– 1.5, – 0.5)
PAC-QOL score, physical discomfort	Baseline	26	2.8 (0.6)	2.8 (2.5, 3.0)	Reference
	24 weeks	19	1.3 (0.9)	1.3 (0.5, 1.8)	– 1.5 (– 2.0, – 1.0)
	48 weeks	17	1.6 (1.1)	1.5 (0.8, 2.0)	– 1.1 (– 1.6, – 0.6)
PAC-QOL score, psychosocial discomfort	Baseline	26	2.2 (0.9)	2.2 (1.5, 3.0)	Reference
	24 weeks	19	0.9 (0.7)	0.9 (0.3, 1.5)	– 1.3 (– 1.8, – 0.8)
	48 weeks	17	1.0 (0.9)	0.6 (0.1, 1.4)	– 1.2 (– 1.8, – 0.7)
PAC-QOL score, worries and concerns	Baseline	26	2.7 (0.8)	2.9 (2.1, 3.3)	Reference
	24 weeks	19	1.3 (1.0)	1.2 (0.5, 2.0)	– 1.4 (– 2.0, – 0.8)
	48 weeks	17	1.4 (1.2)	1.0 (0.5, 1.7)	– 1.3 (– 1.9, – 0.7)
PAC-SYM score, stool symptoms	Baseline	26	2.4 (1.0)	2.6 (2.0, 3.2)	Reference
	24 weeks	18	1.2 (0.7)	1.3 (0.8, 1.6)	– 1.2 (– 1.8, – 0.6)
	48 weeks	17	1.6 (1.0)	1.4 (0.8, 2.4)	– 0.9 (– 1.5, – 0.3)
PAC-SYM score, abdominal symptoms	Baseline	26	2.4 (0.7)	2.4 (2.0, 2.8)	Reference
	24 weeks	18	1.4 (1.0)	1.3 (0.8, 1.8)	– 1.0 (– 1.5, – 0.5)
	48 weeks	17	1.5 (0.8)	1.5 (1.0, 2.0)	– 0.9 (– 1.4, – 0.4)
PAC-SYM score, rectal symptoms	Baseline	26	1.7 (1.0)	1.7 (1.0, 2.0)	Reference
	24 weeks	18	0.8 (0.5)	0.7 (0.3, 1.0)	– 0.9 (– 1.4, – 0.3)
	48 weeks	17	0.8 (1.0)	0.7 (0.3, 1.0)	– 0.9 (– 1.4, – 0.3)
Diary data, bowel frequency, mean no. of attempts to empty bowels	Baseline	22	43.5 (22.0)	45.5 (28.0, 61.0)	Reference
	24 weeks	20	22.9 (18.1)	19.0 (11.5, 25.0)	– 20.5 (– 32.5, – 8.5)
	48 weeks	15	30.6 (16.6)	30.0 (19.0, 44.0)	– 12.9 (– 25.9, 0.1)
Diary data, bowel frequency, mean no. of times stool was actually passed	Baseline	22	27.8 (18.6)	19.5 (15.0, 46.0)	Reference
	24 weeks	21	17.3 (12.2)	14.0 (8.0, 22.0)	– 10.5 (– 20.1, – 0.9)
	48 weeks	15	21.3 (15.3)	19.0 (10.0, 26.0)	– 6.6 (– 17.1, 4.0)
Diary data, nature of bowel movement, mean no. of days laxatives used	Baseline	21	22.3 (6.2)	26.0 (15.0, 28.0)	Reference
	24 weeks	21	23.7 (4.7)	24.0 (21.0, 28.0)	1.4 (– 2.0, 4.7)
	48 weeks	15	22.7 (5.3)	25.0 (18.0, 28.0)	0.4 (– 3.3, 4.1)
Diary data, nature of bowel movement, mean no. of days glycerin suppositories used	Baseline	21	27.7 (0.7)	28.0 (28.0, 28.0)	Reference
	24 weeks	21	26.5 (2.7)	28.0 (26.0, 28.0)	– 1.1 (– 2.2, – 0.0)
	48 weeks	15	27.4 (1.1)	28.0 (27.0, 28.0)	– 0.3 (– 1.5, 0.9)
EQ-VAS scores	Baseline	25	58.6 (18.6)	60.0 (40.0, 75.0)	Reference
	24 weeks	20	73.7 (17.1)	77.0 (60.0, 90.0)	15.1 (4.1, 26.1)
	48 weeks	17	68.2 (19.3)	70.0 (60.0, 80.0)	9.6 (– 1.9, 21.1)
PHQ-9	Baseline	26	8.0 (6.5)	5.0 (4.0, 11.0)	Reference
	24 weeks	18	6.1 (6.0)	4.5 (2.0, 9.0)	– 2.0 (– 6.0, 2.0)
	48 weeks	17	6.7 (7.0)	3.0 (2.0, 10.0)	– 1.3 (– 5.4, 2.7)
GAD-7	Baseline	26	7.1 (6.4)	6.5 (2.0, 10.0)	Reference
	24 weeks	18	5.0 (6.1)	2.5 (0.0, 7.0)	– 2.1 (– 5.9, 1.6)
	48 weeks	17	4.4 (5.7)	2.0 (1.0, 6.0)	– 2.8 (– 6.6, 1.1)
Global patient satisfaction score	Baseline	NA	NA	NA	NA
	24 weeks	18	2.7 (0.8)	3.0 (2.0, 3.0)	NA
	48 weeks	17	2.2 (1.3)	3.0 (1.0, 3.0)	NA
Global patient improvement score	Baseline	NA	NA	NA	NA
	24 weeks	18	72.2 (25.0)	80.0 (67.0, 88.0)	NA
	48 weeks	17	56.5 (34.6)	75.0 (25.0, 80.0)	NA
St Marks Incontinence score	Baseline	26	11.8 (4.7)	13.0 (8.0, 16.0)	Reference
	24 weeks	16	8.7 (4.5)	8.5 (4.5, 13.0)	– 3.1 (– 6.3, 0.1)
	48 weeks	17	8.7 (5.8)	8.0 (3.0, 15.0)	– 3.1 (– 6.2, 0.1)
PISQ-12	Baseline	23	20.5 (6.1)	21.0 (15.0, 25.0)	Reference
	24 weeks	12	18.8 (5.9)	18.0 (15.5, 22.5)	– 1.7 (– 5.7, 2.4)
	48 weeks	12	17.3 (4.5)	17.0 (14.5, 19.0)	– 3.2 (– 7.3, 0.9)
CC-BRQ, avoidance behavior	Baseline	26	45.9 (14.2)	45.5 (32.0, 59.0)	Reference
	24 weeks	18	31.6 (15.1)	26.5 (20.0, 47.0)	– 14.3 (– 23.3, – 5.4)
	48 weeks	17	33.1 (14.4)	29.0 (23.0, 37.0)	– 12.8 (– 21.9, – 3.8)
CC-BRQ, safety behavior	Baseline	26	53.8 (11.3)	55.0 (45.0, 62.0)	Reference
	24 weeks	18	40.1 (10.0)	38.5 (34.0, 48.0)	– 13.7 (– 20.5, – 7.0)
	48 weeks	17	40.9 (11.6)	39.0 (34.0, 44.0)	– 13.0 (– 19.8, – 6.1)
BIPQ, negative perceptions	Baseline	26	39.2 (8.1)	39.0 (33.0, 46.0)	Reference
	24 weeks	18	22.9 (15.0)	21.0 (9.0, 37.0)	– 16.3 (– 23.5, – 9.0)
	48 weeks	17	28.6 (12.7)	31.0 (19.0, 38.0)	– 10.5 (– 17.9, – 3.2)
BIPQ, control and coherence	Baseline	25	19.3 (4.4)	20.0 (18.0, 21.0)	Reference
	24 weeks	18	19.9 (6.7)	20.5 (17.0, 24.0)	0.6 (– 2.7, 3.9)
	48 weeks	16	20.8 (4.7)	21.5 (17.0, 24.5)	1.4 (– 2.0, 4.8)

*SD* standard deviation, *IQR* interquartile range, *CI* confidence interval, *NA* not applicable, *PAC-QOL* Patient Assessment of Constipation Quality of Life, *PAC-SYM* Patient Assessment of Constipation Symptoms, *EQ-VAS* EuroQol Visual Analogue Scale, *PHQ-9* Patient Health Questionnaire-9, *GAD-7* Generalized Anxiety Disorder scale, *PISQ-12* Pelvic Organ Prolapse/Urinary Incontinence Sexual Questionnaire, *CC-BRO* chronic constipation Behavioral Response to Illness Questionnaire, *BIPQ* Brief Illness Perception Questionnaire

The results of the qualitative analysis are shown in Supplementary Table 4.

## Discussion

Our analysis of clinical effectiveness showed a reduced symptom burden and improved disease-specific QoL. The magnitude of the effect of surgery (estimated reduction of 1.09 points in PAC-QoL at 24 weeks) was greater than the minimum clinically important difference sought by design (mean change 1.0 points) and this change was statistically significant. In addition, significant and clinically important improvements in PAC-SYM score, coexistent fecal incontinence, and bowel frequency provided further evidence of the benefit of surgery. The findings of the primary outcome showed a continued improvement for the duration of the study period (estimated 1.38-point reduction in PAC-QoL at 72 weeks), and were supported by a panel of secondary outcome measures, accepting inferential limitations posed by potential attrition bias.

The reduction in anxiety surrounding the use of mesh as time passes and the production of updated consensus guidance on patient selection and operative technique may make further study in this are feasible.

Although some adverse effects were reported, LVMR was safe and well tolerated overall. Patient experience of LVMR was, on the whole, positive. Some patients did not find surgery to be the ‘miracle cure’ that they were looking for and some reported negative experiences in the perioperative period, but for the most part these were not related to the operation itself. Some patients also found benefit from the dietary and behavioral changes that they initiated as a result of advice that they were given as part of the perioperative care package. The mesh controversy dominated staff experience. Developing one nationally recognized information sheet and LVMR surgical certification may assist with patient and surgeon anxieties in future.

Over the last decade, LVMR has become an increasingly popular surgical option for patients with high-grade IRP associated with symptomatic presentation manifest as constipation or incontinence. Table 4 shows data of 12 previous studies published since 2010, 7 of which were prospective [17, 21, 52–56] and 5 retrospective [18, 19, 25, 41, 57] in design. Notable amongst these is the large retrospective cohort study of Consten, et al. [18] which reported outcomes of LVMR in a cohort of 919 patients from 2 centers, with a medium-term follow-up (median, 34 months; range 4 months–12 years). Within the cohort, 677 patients had a main diagnosis of IRP. While some data were unsegregated by baseline phenotype (there were a mix of symptomatic presentations and prolapse type: IRP vs. ERP), the investigators reported resolution of ODS symptoms in approximately 70%. Cumulative risks of mesh complications were low (1.5% after 3, 2.9% after 5, and 4.6% after 10 years), particularly for mesh erosions or infection (1.5% at 10 years), as opposed to cumulative symptom recurrence rates, which were higher as compared to other studies (7.5%, 11.1%, and 14.3% at 3, 5, and 10 years, respectively).

**Table 4:** Studies reporting outcomes of laparoscopic ventral mesh rectopexy (LVMR) in patients with internal rectal prolapse (IRP)

Author	Year	*N*	Design	Median° /Mean°° follow-up, months (range)	Mesh types	Mesh Cx (%)	Mean CCCS°/ODS°°/PAC-SYM°°°		Constipation improved (%)	FISI°/CCIS°°		QoL measures	Anatomical recurrence (%)
							Pre	Post		Pre	Post		
Collinson [17]	2010	75	PCS	12° (3–48)	PP	0	12°	5°	86	28°	8°	NR	5.0
Portier [52]	2011	40^a^	PCS	22°° (6–72)	NR	0	NR	NR	65	13.3°°	3°°	NR	2.5
Sileri [53]	2012	34	PCS	12° (6–30)	B	0	16°	7°	NR	9°	3°	NR	5.9
Formijne Jonkers [19]	2013	157	RCS	30°° (5–83)	PP (varied)	1.3^b^	NR	8.1°	66	NR	NR	NR	2.6
Gosselink [41]	2013	151	RCS	12° (12–12)	NR	NR	2°°°	0.9°°°	NR	24°	12°	PAC-QOL, GIQLI	NR
Borie [25]	2014	52	RCS	NR	PP	NR	16°°	7.6°°	NR	NR	NR	NR	NR
Franceschilli [54]	2015	100	PCS	20° (6–54)	B	0	18.4°	5.4°	92	8.4°	3.3°	NR	14.0
Gosselink [21]	2015	50	PCS	12° (12–12)	PP	0	NR	NR	NR	42°	25°	GIQLI	6
Consten [18]	2015	677	RCH	33.9° (0.4–144)	PP (varied)	4.6^c^	NR	NR	74	NR	NR	NR	14.2^c^
Tsunoda [55]	2016	25	PCS	26° (12–42)	PP	0	11°	5°	59	30°	8°	SF-36, FIQL, PAC-QOL	4.0
Tsunoda [52]	2018	34	PCS	40° (15–58)	PP	2.9	12°	5°	59	30°	14.5°	SF-36	2.9
Degasperi [57]	2020	50	RCH	16.5° (10–44.3)	PP	0	14°	11°	70	NR	NR	SF-36	0

*Cx* complications, *CCCS* Cleveland Clinic constipation score, *ODS* Obstructed Defecation Syndrome score, *PAC-SYM* Patient Assessment of Constipation Symptoms, *FISI* Fecal Incontinence Severity Index, *CCIS* Cleveland Clinic fecal incontinence score, *PP* polypropylene, *B* biologic, *NR* not recorded, *PCS* prospective case series, *RCS* retrospective case series, *RCH* retrospective cohort study, *PAC-QOL* Patient Assessment of Constipation Quality of Life, *GIQLI* Gastrointestinal Quality of Life Index, *SF-36* Short-Form 36 Health Survey, *FIQL* Fecal Incontinence Quality of Life scale

^a^Included 23 open and 17 LVMR

^b^Calculated on a total cohort of 233 patients including indications for LVMR other than IRP

^c^Kaplan–Meier estimate at 10 years of follow-up

In contrast to nearly all previous studies (Table 4), we explored disease-specific QoL using two validated instruments. Tsunoda et al. [55] used validated instruments (Short-Form 36 Health Survey [SF-36], Fecal Incontinence QoL scale [FIQL], and Patient Assessment of Constipation-QoL [PAC-QOL]) to assess quality of life after LVMR in 25 patients with IRP (all females) and 19 with ERP. Compared to the preoperative assessment, almost all the scale scores on the three quality of life instruments significantly improved over time. Gosselink, et al. [41] compared the functional results of LVMR for obstructed defecation secondary to high-grade IRP in 109 patients with normal and 42 with delayed colonic transit. Although preoperative PAC-QOL scores were higher (worse) in the latter group, the total PAC-QOL score was significantly improved in both groups at 12 months (*p* < 0.001). The Gastrointestinal Quality of Life Index (GIQLI) was also improved in both groups. The same authors showed equivalent GIQLI improvements in a series of 50 incontinent patients undergoing LVMR for high-grade IRP (*p* = 0.01) [21].

### Limitations

Several limitations associated with our RCT warrant mention. The study was severely hampered by under-recruitment (28 out of a planned 114 patients). The media scrutiny of the use of mesh undoubtedly affected both patient and surgeon perception and willingness to take part in the study. Some centers paused or abandoned LVMR totally in the light of the mesh controversy, and there was a perception that for others the heightened scrutiny of practice in the protocol also negatively affected recruitment. Difficulties in attracting centers to recruit pre-dated the zenith of the mesh publicity and also reflected wide variation in practice across the UK in terms of both patient selection and LVMR operative technique. The attention we paid to strict inclusion and exclusion criteria (based on guidance from the Pelvic Floor Society, London, UK) [36] led to increased scrutiny in many centres where such surgery was previously being undertaken without rigorous application of these criteria. This regrettably led to a rapid revision (manifest as a drop-off) in the number of patients eligible for recruitment to the trial. With the media storm blowing up and the Cumberlege report in preparation [58], there was never a time when the results of this trial were more needed.

Included patients had a high symptom burden and long duration of symptoms that had been refractory to previous treatments, including a minimum of bowel habit training by a specialist practitioner. Patients had been thoroughly investigated and, therefore, could be considered both ‘hard to treat’ and ‘carefully selected’ for surgical intervention.

However, despite these setbacks, the main aim of the trial, namely to determine the effect size of surgery for the first time in a high-quality experimental design, and thus improve on the level IV evidence provided by 18 case series (as outlined in our systematic review) [23], was addressed, albeit at a lower than desirable level of statistical power.

## Conclusions

Our results show substantial symptomatic benefit (more than we sought by design) to a cohort of highly selected patients from LVMR performed to a standardized technique.

## Supplementary Information

Below is the link to the electronic supplementary material.

Supplementary file1 (DOCX 42 KB)

## References

[1] Collinson R, Cunningham C, D'Costa H (2009). Rectal intussusception and unexplained faecal incontinence: findings of a proctographic study. Colorectal Dis.

[2] Dvorkin LS, Knowles CH, Scott SM (2005). Rectal intussusception: characterization of symptomatology. Dis Colon Rectum.

[3] Grossi U, Heinrich H, Di Tanna GL (2021). Systematic characterization of defecographic abnormalities in a consecutive series of 827 patients with chronic constipation. Dis Colon Rectum.

[4] Grossi U, Di Tanna GL, Heinrich H (2018). Systematic review and meta-analysis: defecography should be a first-line diagnostic modality in patients with refractory constipation. Aliment Pharmacol Ther.

[5] Shorvon PJ, McHugh S, Diamant NE (1989). Defecography in normal volunteers: results and implications. Gut.

[6] Marti MC, Roche B, Deleaval JP (1999). Rectoceles: value of videodefaecography in selection of treatment policy. Colorectal Dis.

[7] Abramov Y, Gandhi S, Goldberg RP (2005). Site-specific rectocele repair compared with standard posterior colporrhaphy. Obstet Gynecol.

[8] Sung VW, Rardin CR, Raker CA (2012). Changes in bowel symptoms 1 year after rectocele repair. Am J Obstet Gynecol.

[9] Guzman Rojas R, Kamisan Atan I, Shek KL (2015). Defect-specific rectocele repair: medium-term anatomical, functional and subjective outcomes. Aust N Z J Obstet Gynaecol.

[10] Boccasanta P, Venturi M, Salamina G (2004). New trends in the surgical treatment of outlet obstruction: clinical and functional results of two novel transanal stapled techniques from a randomised controlled trial. Int J Colorectal Dis.

[11] Renzi A, Brillantino A, Di Sarno G (2011). Improved clinical outcomes with a new contour-curved stapler in the surgical treatment of obstructed defecation syndrome: a mid-term randomized controlled trial. Dis Colon Rectum.

[12] Goede AC, Glancy D, Carter H (2011). Medium-term results of stapled transanal rectal resection (STARR) for obstructed defecation and symptomatic rectal-anal intussusception. Colorectal Dis.

[13] Wong MT, Abet E, Rigaud J (2011). Minimally invasive ventral mesh rectopexy for complex rectocoele: impact on anorectal and sexual function. Colorectal Dis.

[14] Mantoo S, Podevin J, Regenet N (2013). Is robotic-assisted ventral mesh rectopexy superior to laparoscopic ventral mesh rectopexy in the management of obstructed defaecation?. Colorectal Dis.

[15] Knowles CH, Grossi U, Horrocks EJ (2017). Surgery for constipation: systematic review and practice recommendations: graded practice and future research recommendations. Colorectal Dis.

[16] Berman IR (1992). Sutureless laparoscopic rectopexy for procidentia. Technique and implications. Dis Colon Rectum.

[17] Collinson R, Wijffels N, Cunningham C (2010). Laparoscopic ventral rectopexy for internal rectal prolapse: short-term functional results. Colorectal Dis.

[18] Consten EC, van Iersel JJ, Verheijen PM (2015). Long-term outcome after laparoscopic ventral mesh rectopexy: an observational study of 919 consecutive patients. Ann Surg.

[19] Formijne Jonkers HA, Poierrie N, Draaisma WA (2013). Laparoscopic ventral rectopexy for rectal prolapse and symptomatic rectocele: an analysis of 245 consecutive patients. Colorectal Dis.

[20] Slawik S, Soulsby R, Carter H (2008). Laparoscopic ventral rectopexy, posterior colporrhaphy and vaginal sacrocolpopexy for the treatment of recto-genital prolapse and mechanical outlet obstruction. Colorectal Dis.

[21] Gosselink MP, Joshi H, Adusumilli S (2015). Laparoscopic ventral rectopexy for faecal incontinence: equivalent benefit is seen in internal and external rectal prolapse. J Gastrointest Surg.

[22] Borie F, Coste T, Bigourdan JM (2016). Incidence and surgical treatment of synthetic mesh-related infectious complications after laparoscopic ventral rectopexy. Tech Coloproctol.

[23] Grossi U, Knowles CH, Mason J (2017). Surgery for constipation: systematic review and practice recommendations: Results II: hitching procedures for the rectum (rectal suspension). Colorectal Dis.

[24] Badrek-Amoudi AH, Roe T, Mabey K (2013). Laparoscopic ventral mesh rectopexy in the management of solitary rectal ulcer syndrome: a cause for optimism?. Colorectal Dis.

[25] Borie F, Bigourdan JM, Pissas MH (2014). Laparoscopic ventral rectopexy for the treatment of outlet obstruction associated with recto-anal intussusception and rectocele: a valid alternative to STARR procedure in patients with anal sphincter weakness. Clin Res Hepatol Gastroenterol.

[26] van den Esschert JW, van Geloven AA, Vermulst N (2008). Laparoscopic ventral rectopexy for obstructed defecation syndrome. Surg Endosc.

[27] Hasan HM, Hasan HM (2012) Stapled transanal rectal resection for the surgical treatment of obstructed defecation syndrome associated with rectocele and rectal intussusception. ISRN Surg 2012:652345. 10.5402/2012/652345 10.5402/2012/652345 PMC3346690 22577584

[28] Jayne DG, Schwandner O, Stuto A (2009). Stapled transanal rectal resection for obstructed defecation syndrome: one-year results of the European STARR Registry. Dis Colon Rectum.

[29] Knowles CH, Dinning PG, Pescatori M (2009). Surgical management of constipation. Neurogastroenterol Motil.

[30] Rubin R (2019). Mesh implants for women: scandal or standard of care?. JAMA.

[31] Grossi U, Stevens N, McAlees E (2018). Stepped-wedge randomised trial of laparoscopic ventral mesh rectopexy in adults with chronic constipation: study protocol for a randomized controlled trial. Trials.

[32] Knowles CH, Booth L, Brown SR (2021). Non-drug therapies for the management of chronic constipation in adults: the CapaCiTY research programme including three RCTs. Progr Grant Appl Res.

[33] Tack J, Muller-Lissner S (2009). Treatment of chronic constipation: current pharmacologic approaches and future directions. Clin Gastroenterol Hepatol.

[34] Norton C, Emmanuel A, Stevens N (2017). Habit training versus habit training with direct visual biofeedback in adults with chronic constipation: study protocol for a randomised controlled trial. Trials.

[35] Palit S, Bhan C, Lunniss PJ (2014). Evacuation proctography: a reappraisal of normal variability. Colorectal Dis.

[36] Mercer-Jones MA, Brown SR, Knowles CH (2017). Position Statement by The Pelvic Floor Society on behalf of The Association of Coloproctology of Great Britain and Ireland on the use of mesh in ventral mesh rectopexy (VMR). Colorectal Dis.

[37] Mercer-Jones MA, D'Hoore A, Dixon AR (2014). Consensus on ventral rectopexy: report of a panel of experts. Colorectal Dis.

[38] Coleman M, Cecil T (2017) Laparoscopic colorectal surgery: the Lapco Manual

[39] Powar MP, Ogilvie JW, Stevenson AR (2013). Day-case laparoscopic ventral rectopexy: an achievable reality. Colorectal Dis.

[40] Marquis P, De La Loge C, Dubois D (2005). Development and validation of the Patient Assessment of Constipation Quality of Life questionnaire. Scand J Gastroenterol.

[41] Gosselink MP, Adusumilli S, Harmston C (2013). Impact of slow transit constipation on the outcome of laparoscopic ventral rectopexy for obstructed defaecation associated with high grade internal rectal prolapse. Colorectal Dis.

[42] Dubois D, Gilet H, Viala-Danten M (2010). Psychometric performance and clinical meaningfulness of the Patient Assessment of Constipation-Quality of Life questionnaire in prucalopride (RESOLOR) trials for chronic constipation. Neurogastroenterol Motil.

[43] Yiannakou Y, Tack J, Piessevaux H (2017). The PAC-SYM questionnaire for chronic constipation: defining the minimal important difference. Aliment Pharmacol Ther.

[44] Spitzer RL, Kroenke K, Williams JB (2006). A brief measure for assessing generalized anxiety disorder: the GAD-7. Arch Intern Med.

[45] Kroenke K, Spitzer RL, Williams JB (2001). The PHQ-9: validity of a brief depression severity measure. J Gen Intern Med.

[46] Vaizey CJ, Carapeti E, Cahill JA (1999). Prospective comparison of faecal incontinence grading systems. Gut.

[47] Rogers RG, Coates KW, Kammerer-Doak D (2003). A short form of the Pelvic Organ Prolapse/Urinary Incontinence Sexual Questionnaire (PISQ-12). Int Urogynecol J Pelvic Floor Dysfunct.

[48] Spence M, Moss-Morris R, Chalder T (2005). The Behavioural Responses to Illness Questionnaire (BRIQ): a new predictive measure of medically unexplained symptoms following acute infection. Psychol Med.

[49] Broadbent E, Petrie KJ, Main J (2006). The brief illness perception questionnaire. J Psychosom Res.

[50] Herdman M, Gudex C, Lloyd A (2011). Development and preliminary testing of the new five-level version of EQ-5D (EQ-5D-5L). Qual Life Res.

[51] Frank L, Kleinman L, Farup C (1999). Psychometric validation of a constipation symptom assessment questionnaire. Scand J Gastroenterol.

[52] Portier G, Kirzin S, Cabarrot P (2011). The effect of abdominal ventral rectopexy on faecal incontinence and constipation in patients with internal intra-anal rectal intussusception. Colorectal Dis.

[53] Sileri P, Franceschilli L, de Luca E (2012). Laparoscopic ventral rectopexy for internal rectal prolapse using biological mesh: postoperative and short-term functional results. J Gastrointest Surg.

[54] Franceschilli L, Varvaras D, Capuano I (2015). Laparoscopic ventral rectopexy using biologic mesh for the treatment of obstructed defaecation syndrome and/or faecal incontinence in patients with internal rectal prolapse: a critical appraisal of the first 100 cases. Tech Coloproctol.

[55] Tsunoda A, Takahashi T, Ohta T (2016). Quality of life after laparoscopic ventral rectopexy. Colorectal Dis.

[56] Tsunoda A, Takahashi T, Hayashi K (2018). Laparoscopic ventral rectopexy in patients with fecal incontinence associated with rectoanal intussusception: prospective evaluation of clinical, physiological and morphological changes. Tech Coloproctol.

[57] Degasperi S, Scarpa M, Zini O (2020). Laparoscopic ventral rectopexy for obstructed defecation: functional results and quality of life. Surg Laparosc Endosc Percutan Tech.

[58] https://www.immdsreview.org.uk/downloads/IMMDSReview_Web.pdf. Accessed 23 Nov 2021

