# Supplementary material for: Stepped-wedge randomized controlled trial of laparoscopic ventral mesh rectopexy in adults with chronic constipation
Source: Tech Coloproctol. 2022 May 19;26(12):941–52. doi: 10.1007/s10151-022-02633-w (PMC9117980; doi:10.1007/s10151-022-02633-w)
Supplement: Supplementary file 1 — Supplementary file1 (DOCX 42 KB) [file 10151_2022_2633_MOESM1_ESM.docx]

**Supplementary Table 1.** Surgical quality assessment

| *Planned monitoring* |
| --- |
| Potential principal investigators had to record and submit two unedited and anonymised videos of laparoscopic ventral mesh rectopexy (LVMR) performed in non-study patients. Each video was allocated to two peer reviewers of a three-member expert panel. Based on blinded assessment of unedited and anonymised videos by expert review, the panel then decided whether or not the principal investigator was ‘adherent’ to the standardised technique. Any disagreement was resolved by consensus after consulting a third independent expert. If deemed ‘nonadherent’ to the standardised technique, the site was notified that a step needed to be corrected and invited to submit another video for similar review. An unedited video of the first patient at each site enrolled in CapaCiTY trial 3 was also reviewed in this manner. Any ‘failure’ to comply with the standardised surgical technique for LVMR in any submitted video of a study patient would trigger the request for a further submission of the next recruited patient from that centre. A second judgement of ‘non-adherence’ to the standardised technique would trigger an onsite training and monitoring session for the site. Monitoring would continue until adherence was achieved. A third ‘non-adherence’ or ‘failure’ would result in withdrawal of the site/principal investigator from the trial. |
| *Random monitoring* |
| All principal investigators had to record and submit the unedited and anonymised video of the LVMR performed in a randomly selected patient enrolled in CapaCiTY trial 3 (one in five at site level).The adherence to the standardised technique was established by consensus as described for the planned monitoring. |
| *Triggered monitoring* |
| The Data Monitoring Committee reviewed the morbidity and mortality rates and adverse events and serious adverse events from all sites. Safety concerns could trigger additional monitoring or onsite training and mentorship visits to take place by expert panel. Repeated ‘non adherence’ or ‘failure’ to comply would result in withdrawal of the site/principal investigator from the trial. |

**Supplementary Table 2.** The CapaCiTY trial 3 outcome measure data at baseline

| **Outcome measure** | **Group 1:**  **LVMR performed at T0**  **(N = 9)** | **Group 2:**  **LVMR performed at T12**  **(N = 10)** | **Group 3:**  **LVMR performed at T24**  **(N = 9)** |
| --- | --- | --- | --- |
| PAC-QOL score (points) |  |  |  |
| Overall, mean (SD) | 2.7 (0.6) | 2.7 (0.6) | 2.5 (0.8) |
| Missing, n (%) | 1 (11.1) | 0 (0.0) | 1 (11.1) |
| Dissatisfaction, mean (SD) | 3.1 (0.6) | 3.1 (0.4) | 3.2 (0.9) |
| Missing, n (%) | 1 (11.1) | 0 (0.0) | 1 (11.1) |
| Physical discomfort, mean (SD) | 2.7 (0.6) | 2.9 (0.6) | 2.7 (0.5) |
| Missing, n (%) | 1 (11.1) | 0 (0.0) | 1 (11.1) |
| Psychosocial discomfort, mean (SD) | 2.3 (0.9) | 2.3 (0.9) | 2.1 (0.9) |
| Missing, n (%) | 1 (11.1) | 0 (0.0) | 1 (11.1) |
| Worries and concerns, mean (SD) | 2.7 (0.7) | 2.8 (0.7) | 2.5 (1.0) |
| Missing, n (%) | 1 (11.1) | 0 (0.0) | 1 (11.1) |
| PAC-SYM score (points) |  |  |  |
| Overall, mean (SD) | 2.3 (0.4) | 2.1 (0.6) | 2.3 (0.7) |
| Missing, n (%) | 1 (11.1) | 1 (10.0) | 0 (0.0) |
| Stool symptoms, mean (SD) | 2.7 (0.7) | 2.1 (0.9) | 2.5 (1.3) |
| Missing, n (%) | 1 (11.1) | 1 (10.0) | 0 (0.0) |
| Abdominal symptoms, mean (SD) | 2.3 (0.6) | 2.5 (0.8) | 2.4 (0.8) |
| Missing, n (%) | 1 (11.1) | 1 (10.0) | 0 (0.0) |
| Rectal symptoms, mean (SD) | 1.5 (0.7) | 1.6 (1.2) | 2.0 (1.2) |
| Missing, n (%) | 1 (11.1) | 1 (10.0) | 0 (0.0) |
| Bowel frequency, number reported over 14 days (diary data) |  |  |  |
| Attempts to empty bowels, mean (SD) | 41.3 (16.2) | 43.9 (27.6) | 45.1 (23.2) |
| Missing, n (%) | 2 (22.2) | 2 (20.0) | 2 (22.2) |
| Times stool was actually passed, mean (SD) | 21.3 (14.5) | 36.5 (18.7) | 24.4 (20.8) |
| Missing, n (%) | 2 (22.2) | 2 (20.0) | 2 (22.2) |
| Nature of bowel movement, number of days out of 14 (diary data) |  |  |  |
| Laxatives used, mean (SD) | 20.4 (5.7) | 24.1 (6.6) | 22.3 (6.6) |
| Missing, n (%) | 2 (22.2) | 3 (30.0) | 2 (22.2) |
| Glycerine suppositories used, mean (SD) | 27.7 (0.8) | 27.7 (0.8) | 27.6 (0.8) |
| Missing, n (%) | 2 (22.2) | 3 (30.0) | 2 (22.2) |
| EQ-5D-5L ‘no problem’ indicated, n (%) |  |  |  |
| Mobility | 4 (44.4) | 2 (20.0) | 4 (44.4) |
| Missing | 2 (22.2) | 1 (10.0) | 0 (0.0) |
| Self-care | 1 (11.1) | 1 (10.0) | 1 (11.1) |
| Missing | 2 (22.2) | 1 (10.0) | 0 (0.0) |
| Usual activities | 5 (55.6) | 7 (70.0) | 6 (66.7) |
| Missing | 2 (22.2) | 1 (10.0) | 0 (0.0) |
| Pain/discomfort | 7 (77.8) | 9 (90.0) | 9 (100.0) |
| Missing | 2 (22.2) | 1 (10.0) | 0 (0.0) |
| Anxiety/depression | 6 (66.7) | 4 (40.0) | 6 (66.7) |
| Missing | 2 (22.2) | 1 (10.0) | 0 (0.0) |
| EQ-VAS score (points) |  |  |  |
| Total, mean (SD) | 59.3 (14.3) | 53.4 (23.0) | 63.3 (17.3) |
| Missing, n (%) | 2 (22.2) | 1 (10.0) | 0 (0.0) |
| PHQ 9 depression severity, n (%) |  |  |  |
| None | 5 (55.6) | 2 (20.0) | 3 (33.3) |
| Mild | 1 (11.1) | 4 (40.0) | 3 (33.3) |
| Moderate | 2 (22.2) | 1 (10.0) | 1 (11.1) |
| Moderately severe | 0 (0.0) | 0 (0.0) | 2 (22.2) |
| Severe | 0 (0.0) | 2 (20.0) | 0 (0.0) |
| Missing | 1 (11.1) | 1 (10.0) | 0 (0.0) |
| GAD 7 anxiety severity, n (%) |  |  |  |
| None | 4 (44.4) | 4 (40.0) | 4 (44.4) |
| Mild | 3 (33.2) | 1 (10.0) | 3 (33.3) |
| Moderate | 0 (0.0) | 2 (20.0) | 1 (11.1) |
| Severe | 1 (11.1) | 2 (20.0) | 1 (11.1) |
| Missing | 1 (11.1) | 1 (10.0) | 0 (0.0) |
| St Marks Incontinence score (points) |  |  |  |
| Total, mean (SD) | 12.4 (3.6) | 11.7 (5.9) | 11.3 (4.7) |
| Missing, n (%) | 1 (11.1) | 0 (0.0) | 1 (11.1) |
| PISQ-12 score (points) |  |  |  |
| Total, mean (SD) | 19.3 (7.1) | 21.4 (6.7) | 20.8 (5.4) |
| Missing, n (%) | 2 (22.2) | 3 (30.0) | 0 (0.0) |
| The chronic constipation behavioural responses questionnaire - CC-BRQ score |  |  |  |
| Avoidance behaviour, mean (SD) | 51.4 (13.1) | 42.4 (13.1) | 44.6 (16.2) |
| Missing, n (%) | 1 (11.1) | 1 (10.0) | 0 (0.0) |
| Safety behaviour, mean (SD) | 51.8 (12.9) | 50.7 (11.4) | 58.9 (8.8) |
| Missing, n (%) | 1 (11.1) | 1 (10.0) | 0 (0.0) |
| Brief illness perception questionnaire - BIPQ (CC) score |  |  |  |
| Negative perceptions, mean (SD) | 40.7 (6.9) | 38.9 (7.6) | 38.1 (10.1) |
| Missing, n (%) | 1 (11.1) | 1 (10.0) | 0 (0.0) |
| Control and coherence subscale, mean (SD) | 18.9 (5.1) | 17.9 (4.1) | 21.1 (4.0) |
| Missing, n (%) | 2 (22.2) | 1 (10.0) | 0 (0.0) |

Owing to the lack of male participants, male sexual health outcomes are not summarized in this report.

PAC-QOL:Patient Assessment of Constipation Quality of Life ; PAC-SYM:Patient Assessment of Constipation Symptoms; EQ-VAS: EuroQol Visual Analogue Scale ; PHQ-9: Patient Health Questionnaire-9 ; GAD-7: Generalized Anxiety Disorder scale; PISQ-12 : Pelvic Organ Prolapse/Urinary Incontinence Sexual Questionnaire ; CC-BRO: chronic constipation Behavioral Response to Illness Questionnaire; BIPQ:Brief Illness Perception Questionnaire

**Supplementary Table 3.** Safety analyses

|  | **Group 1:**  **LVMR performed at T0**  **(N = 9)** | **Group 2:**  **LVMR performed at T12**  **(N = 10)** | **Group 3:**  **LVMR performed at T24**  **(N = 9)** |
| --- | --- | --- | --- |
| **ADVERSE EVENTS (AE)** |  |  |  |
| Number of patients reporting AEs | 8 | 7 | 1 |
| Number of AEs reported by category | 12 | 17 | 1 |
| abdominal pain | 2 | 2 | 0 |
| bloating | 0 | 1 | 0 |
| constipation | 1 | 0 | 0 |
| haemorrhoids | 1 | 0 | 0 |
| loose motions | 0 | 1 | 0 |
| vaginal / perineal bulging | 2 | 1 | 0 |
| anal / rectal pain or discomfort | 1 | 3 | 0 |
| rectal bleeding | 1 | 1 | 0 |
| miscellaneous | 4 | 8 | 0 |
| Severity |  |  |  |
| mild | 5 | 5 | 0 |
| moderate | 7 | 12 | 1 |
| Causality |  |  |  |
| unlikely related | 3 | 4 | 1 |
| possibly related | 4 | 7 | 0 |
| definitely related | 3 | 6 | 0 |
| Action |  |  |  |
| no action taken | 8 | 7 | 1 |
| withdrawal | 1 | 0 | 0 |
| concomitant medication | 2 | 9 | 0 |
| non-drug therapy | 1 | 0 | 0 |
| hospitalization | 0 | 1 | 0 |
| Outcome* |  |  |  |
| unresolved | 4 | 6 | 1 |
| resolving | 3 | 2 | 0 |
| resolved | 5 | 9 | 0 |
| **SERIOUS ADVERSE EVENTS (SAE)** |  |  |  |
| Number of patients reporting serious adverse events | 2 | 3 | 0 |
| Causality  unlikely related | 1 | 0 | 0 |
| possibly related | 0 | 2 | 0 |
| definitely related | 1 | 1 | 0 |
| Expectedness |  |  |  |
| expected | 1 | 2 | 0 |
| unexpected | 1 | 1 | 0 |
| Action |  |  |  |
| no action taken | 0 | 1 | 0 |
| concomitant medication | 1 | 1 | 0 |
| non-drug therapy | 1 | 0 | 0 |
| hospitalization | 0 | 1 | 0 |
| Outcome** |  |  |  |
| unresolved | 1 | 0 | 0 |
| resolved | 1 | 3 | 0 |

* at time of recording AE

** indicated SAE was unresolved at time of reporting

**Supplementary Table 4.** Qualitative analysis

*Patient experience*

Ten participants were interviewed; 9 of them opted for LVMR; 1 was interviewed a year after declining surgery. For some, the decision to undergo surgery was a much-welcomed pragmatic option. Others appeared to go through a longer deliberation process about potential adverse effects of the surgery, including the recently publicized issues around mesh usage in surgery. The health-care professional’s detailed explanation of the surgery, as well as trust in the surgeon and wider clinical team, seemed to mitigate these concerns. For 1 patient, the reasons for not opting for surgery at this time included several current physical and mental health issues and she chose to manage her constipation with a change in diet, transanal irrigation, suppositories and laxatives, as advised by health-care professionals.

A few patients experienced surgical delays, cancellations and pathway difficulties due to administration or cross-departmental communication issues. A number reported a very good hospital stay experience and described how they felt supported by the surgical and wider health-care team. Some patients reported that the surgery was better than they had initially expected. However, a few patients did report negative experiences during their hospital stay. These did not appear to be related to the surgery itself, but rather to the before and after care provided on the wards.

The level of postsurgical pain seemed to vary. For some, pain was not much of an issue, whereas for others postoperative pain was a significant feature. Recovery times also varied, with a couple of patients going back to work within weeks. However, for several it took between 6 weeks and 9 months to fully recover postoperatively.

Some patients described how their bowel functioning was better immediately after surgery. For others, it took a few weeks to start seeing improvement. However, for a few patients the surgery did not seem to improve their constipation. Some patients have reported continued success and a better QoL both mentally and physically and would recommend the surgery to someone who was experiencing similar bowel issues. For others, the surgery has corrected some but not all issues, such as being able to pass stools in a timely manner. A few patients reported that, although surgery initially seemed to be addressing their problematic bowel issues, they were now re-experiencing these problems. Some had new defecation pain; others were concerned about whether mesh use played a role in continued pain following surgery.

*Staff experience*

Eight staff members (3 surgeons, 3 research nurses, 1 assistant medical professional and 1 research team member) were also interviewed. Because of the media coverage there was reluctance from surgeons to recruit patients for the study and to surgery in general, or surgeons decided to no longer perform LVMR at all. These media concerns have led to surgeons directly expressing anxieties. In addition, some hospitals erred in overemphasizing the risks involved, providing patients with multiple consent forms, which potentially exacerbated patient anxieties about LVMR. Entire organizations chose to no longer offer the surgery because of litigation concerns.

Surgeons noted that LVMR surgery is technically challenging, particularly if the patient has additional medical complications. Furthermore, owing to the technical nature of the operation, extensive experience of carrying out LVMR surgeries is deemed necessary to perform the procedure, limiting the number of surgeons able to offer this operation.

However, the general sense from the interviewed staff was that LVMR is helpful for most patients, although staff also recognize that some patients (particularly those who have multiple medical issues) may not experience lasting beneficial results.
